# Study on mix proportion and mechanism of fine-grained tailing composite anti-seepage material

**DOI:** 10.1371/journal.pone.0355719

**Published:** 2026-08-11

**Authors:** Zhiyuan Mao, Yu Qiu, Qiangsheng Yang, Weiping Duan

**Affiliations:** 1 Sinosteel Maanshan General Institute Of Mining Reaearch CO., LTD, Ma’anshan, Anhui, People’s Republic of China; 2 HuaweiNational Engineering Research Center for Effcient Recycling of Metallic Mineral Resources Co., Ltd, Ma’anshan, Anhui, People’s Republic of China; 3 State key Laboratory of Metal Mine Mining Safety and Disaster Prevention and Control, Ma’anshan, Anhui, People’s Republic of China; Tribhuvan University, NEPAL

## Abstract

Traditional cement-bentonite cutoff materials suffer weak chemical corrosion resistance, and existing tailing-based barriers mostly take tailing as auxiliary filler, lacking multi-component synergistic modification with fine full tailing as primary aggregate. This study prepares a composite anti-seepage material with full fine tailing as main aggregate mixed with Portland cement, sodium bentonite and sodium silicate. Single-factor controlled variable tests with six groups of specimens were carried out to adjust cement and bentonite dosages; permeability tests, quadruple direct shear tests and Micro-CT scanning were conducted to test hydraulic-mechanical properties and micro-pore structure.Results showed that raw tailing possessed a permeability coefficient of 5.3 × 10 ^₋4^ cm/s while modified samples range from 1.1 × 10 ^₋^ ⁷ to 5.5 × 10 ^₋^ ⁷ cm/s. With bentonite fixed at 8 kg, increasing cement dosage raises permeability but greatly lifts shear strength: cohesion rises from 63.2 kPa to 345.2 kPa and internal friction angle grows from 30.2° to 39.1°. With cement fixed at 20 kg, rising bentonite content continuously reduces permeability to 1.1 × 10 ^₋^ ⁷ cm/s and slightly improves strength to 182.2 kPa cohesion and 38.9° friction angle. CT results prove the optimal specimen only has 1.94% porosity, controlled by cement hydration C-S-H gel cementation and bentonite swelling pore-blocking. This material realizes large-scale tailing recycling, fills the research gap of tailing-dominated composite barriers, and provides technical support for tailing pond vertical anti-seepage engineering with remarkable environmental and economic benefits.

## 1. Introduction

As a core anti-seepage and pollutant interception measure for tailings ponds, contaminated sites and hydraulic projects, vertical cutoff walls have been widely applied globally. The material must simultaneously meet strict requirements of low permeability, sufficient mechanical strength, chemical corrosion resistance and long-term service durability [1–3].

Conventional cement-bentonite (CB) slurry barriers dominate engineering practice due to simple construction, self-hardening behavior and low cost. Their dense impermeable layer forms via the synergistic coagulation and adsorption between cement hydration products and bentonite [4–6]. Nevertheless, under acidic leachate, sulfate and heavy metal contamination, ordinary CB materials suffer elevated permeability, strength degradation and crack propagation, leading to poor chemical compatibility and unstable long-term performance [7–9].

To enhance sustainability, industrial solid waste recycling has become a mainstream research direction for barrier materials. Partial or full replacement of cement with ground granulated blast furnace slag produces slag-CB composites, cutting carbon emissions while boosting anti-corrosion and self-repair capacity [10–12]. Nano plastic waste combined with nano-titania fillers effectively densifies cement matrix and raises compressive strength, establishing a theoretical basis for multi-component nano synergistic modification of cementitious materials [13].

Polymer modification and microcapsule self-healing methods further improve material toughness and dry-wet cycle resistance [14,15]. Meanwhile, alkali-activated geopolymer barriers have developed rapidly: activated by sodium silicate, fly ash/slag/bentonite blended cement-free/low-cement systems deliver outstanding early strength, seepage stability and heavy metal immobilization [16–19]. Industrial kiln waste-based geopolymers further confirm multi-solid-waste alkali-activated gelation, where cement kiln dust and industrial by-products act as precursors to synthesize low-carbon barrier binders [20].

Tailing reutilization represents another vital branch of anti-seepage material research. Iron, gold and coal gangue tailings are widely adopted as aggregates/fillers in cement-bentonite cutoff walls and backfill solidification, reducing solid waste volume and lowering project costs [21–24]. Existing works have optimized mechanical and seepage performance of high-tailing mixtures and evaluated their durability under sulfate and heavy metal erosion [25–26]. In pavement engineering, microwave-modified asphalt using waste tire rubber powder improves long-term aging resistance, with microscopic characterization revealing its modification mechanism [27]. Separately, the same research group developed asymmetric fluorescent liquid crystal molecules for organic optoelectronic sensing, an independent functional material field irrelevant to tailings anti-seepage engineering [28].

Current studies mostly treat tailings as auxiliary fillers or only adopt single cement solidification. Few reports construct multi-component synergistic barrier systems with full fine tailings as the main aggregate, nor clarify the co-cementation mechanism of cement and sodium silicate under high tailings content [29–30]. Different from conventional CB, slag-CB and single geopolymer materials, the tailing-sodium silicate-bentonite-cement composite proposed herein takes tailings as the primary aggregate. Sodium silicate alkali activation elevates early strength, cement provides long-term cementation support, and bentonite optimizes plasticity and barrier capacity, enabling massive tailings consumption. The material maintains permeability below 10 ^₋^ ^8^ cm/s with favorable dry-wet, chemical and heavy metal resistance, featuring higher waste utilization, environmental and economic benefits for complex tailings pond anti-seepage engineering [31,32].

Targeting the above identified research gaps, this study prepares composite anti-seepage materials with full fine tailings as the main aggregate, compounded with Portland cement, sodium bentonite and sodium silicate. Six single-factor variable specimen groups are designed to quantify the influence of cement and bentonite dosage on permeability and shear strength. Permeability tests, quadruple direct shear tests and Micro-CT scanning are carried out to characterize hydraulic-mechanical behaviors and internal pore microstructure. The synergistic modification mechanism coupling cement hydration gelation, bentonite swelling plugging and sodium silicate alkali activation is elaborated, alongside the quantitative correlation between raw material dosage, pore structure and macro engineering performance. The optimal material mix proportion is determined, providing technical guidance for tailings pond vertical cutoff wall construction and realizing high-value resource recovery of mine tailings solid waste.

## 2. Materials and methods

### 2.1. Raw experimental materials

**Fig 1:**
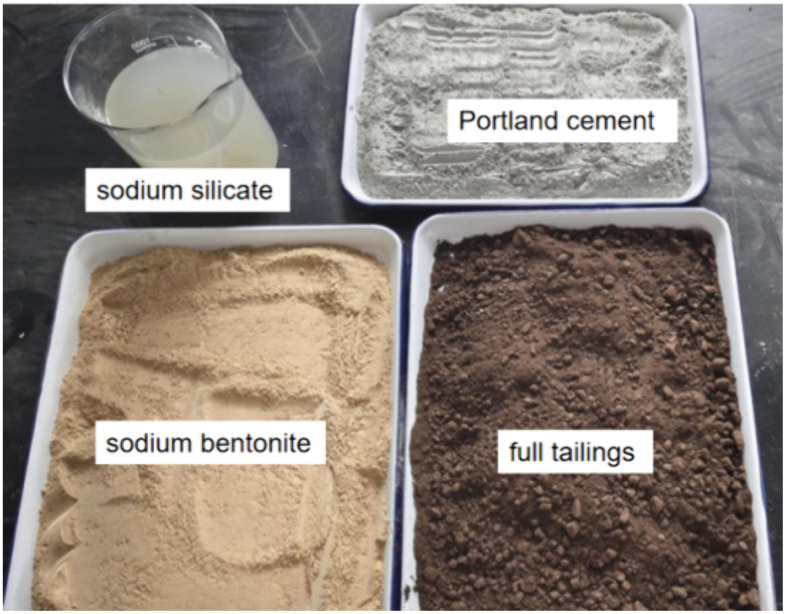
Experimental materials.

The raw materials adopted in this study consist of full fine tailing, ordinary Portland cement, sodium bentonite and sodium silicate, as displayed in Fig 1.

**Fig 2:**
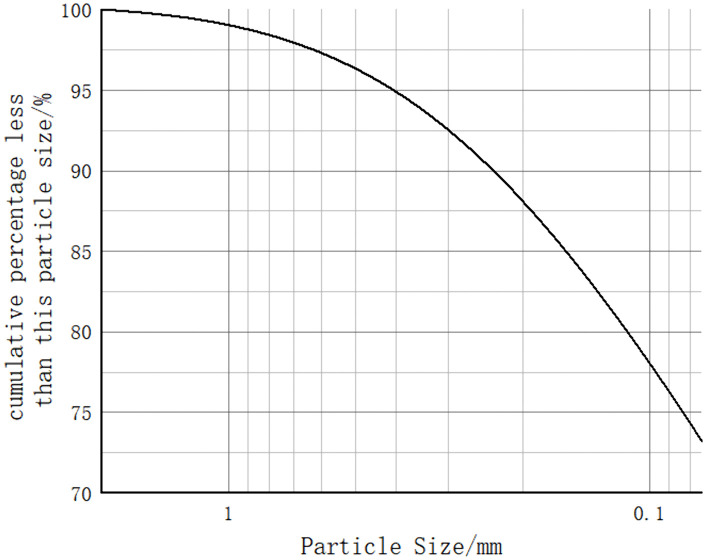
The particle size gradation curve of tailing.

The on-site collected tailing were oven-dried prior to testing. The raw tailing possess a natural water content of 12.6%, bulk density of 1.03 g·cm ^₋^ ^3^, specific gravity of 3.02 and void ratio of 2.301. The basic physical indices are summarized in Table 1, while the particle size distribution is listed in Table 2. According to the grading data, the tested material is classified as silty tailing. The particle size distribution curve of tailing is plotted in Fig 2.

**Table 1:** Physical Properties of tailing Specimen.

w(%)	ρ(g/cm^3^)	ρ_d(_g/cm^3^)	Gs	e	I_p_(%)
12.6	1.03	0.91	3.02	2.301	8.6

**Table 2:** Particle size range.

Particle size Range(mm)	2 ~ 0.5	0.5 ~ 0.25	0.25 ~ 0.075	<0.075
Percentage(%)	0.9	4.9	21.0	73.2

Type 42.5R Portland cement was adopted. The core mineral composition and mass fraction of Type 42.5R Portland cement are as follows: tricalcium silicate (C₃S) accounts for 50%–60%, dicalcium silicate (C₂S) for 15%–25%, tricalcium aluminate (C₃A) for 7%–15%, and tetracalcium aluminoferrite (C₄AF) for 10%–18%. The total mass fraction of these four minerals exceeds 95%, with the remaining small portion consisting of minor components such as free calcium oxide, free magnesium oxide, and sulfur trioxide.

The corresponding chemical composition and its mass fraction range are as follows: calcium oxide (CaO) 62%–67%, silicon dioxide (SiO₂) 20%–24%, aluminium oxide (Al₂O₃) 4%–7%, iron(III) oxide (Fe₂O₃) 2.5%–6%. In addition, the content of sulfur trioxide (SO₃) did not exceed 3.5%, magnesium oxide (MgO) shall not exceed 5.0%, and the alkali content (expressed as Na₂O equivalent) shall not exceed 0.6%–1.0%. Among these components, tricalcium silicate (C₃S) and tricalcium aluminate (C₃A), which have relatively high contents, are the key components ensuring its early strength performance.

Bentonite is generally categorized into sodium and calcium types. During mineral formation or artificial modification, exchangeable cations (Na⁺ or Ca^2+^) attach to the surface of clay mineral particles. Sodium bentonite exhibits prominent hydration capacity: weakly bound Na⁺ cations readily dissociate and adsorb abundant water molecules, triggering interlayer expansion of montmorillonite crystals to form a continuous low-permeability barrier. In comparison, Ca^2+^ in calcium bentonite bears strong electrostatic binding force, which limits water infiltration and leads to poor particle dispersibility and weak impermeability. Considering its outstanding swelling and anti-seepage properties, sodium bentonite was selected as the raw material in this work.

**Fig 3:**
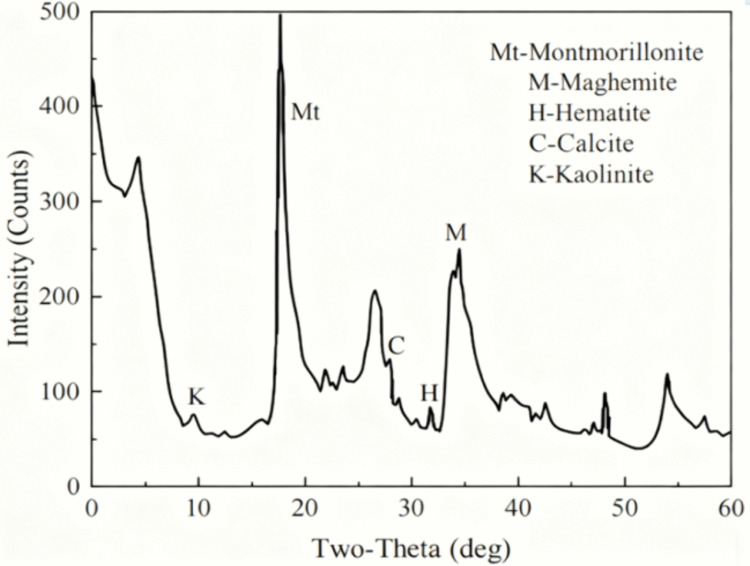
XRD of sodium bentonite.

X-ray diffraction (XRD) patterns (Fig 3) showed that montmorillonite constituted the primary mineral phase of sodium bentonite. The spectrum exhibits diffraction peaks of maghemite, hematite, calcite and kaolinite, with relatively weak diffraction intensity. According to the results of methylene blue adsorption test, the content of montmorillonite in the bentonite is approximately 58.2%.

### 2.2. Sample mix proportion design

**Fig 4:**
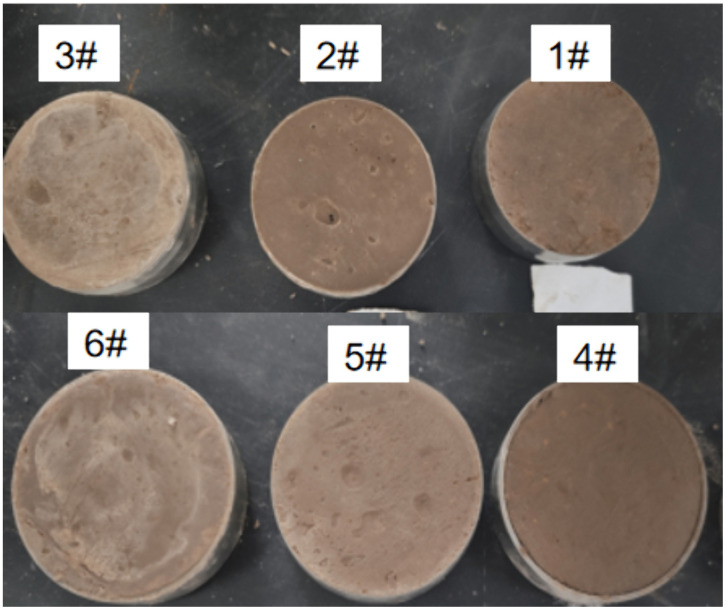
Photographs of prepared specimens.

A total of six samples were prepared, with Sample 1# serving as the raw tailing control. The mix ratios of the test samples are shown in Table 3, and the photos of the samples are presented in Fig 4. A single-factor controlled variable test design was employed to determine the optimal mix ratio of the anti-seepage material, keeping the tailing mass constant while varying the cement and bentonite dosages.

**Table 3:** Raw material mass of each test specimen.

Sample ID	full tailing/kg (percentage)	Portland cement/kg (percentage)	sodium bentonite/kg (percentage)	sodium silicate/kg (percentage)	water/kg (percentage)
1#	30				
	(100%)				
2#	30	20	8	3	35
	(31.25%)	(20.83%)	(8.33%)	(3.13%)	(36.46%)
3#	30	30	8	3	35
	(28.30%)	(28.30%)	(7.55%)	(2.83%)	(33.02%)
4#	30	10	8	3	35
	(34.88%)	(11.63%)	(9.30%)	(3.49%)	(40.70%)
5#	30	20	16	3	35
	(28.85%)	(19.23%)	(15.38%)	(2.88%)	(33.65%)
6#	30	20	24	3	35
	(26.79%)	(17.86%)	(21.43%)	(2.68%)	(31.25%)

### 2.3. Test methods

#### 2.3.1. Permeation test.

**Fig 5:**
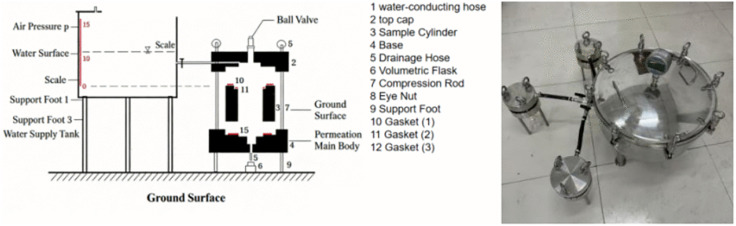
Schematic diagram of permeability test apparatus.

The extremely low permeability coefficient of anti-seepage materials makes it difficult to measure sample permeability within a short time using the conventional falling-head test. To accelerate testing, the permeability test employed a specialized instrument (patented by Suzhou Shuihuan Geotechnical Technology Co., Ltd.) — An Experimental Device for Measuring the Permeability of Fine-grained Soil. The model of the instrument is shown in Fig 5. By applying pressure to the water head, this instrument can quickly determine the permeability coefficient of low-permeability materials.

After soil samples are obtained with a ring knife that is 4 cm in height and 30 cm^2^ in cross-sectional area for the experiment, the calculation formula for the permeability coefficient is as follows:


$$\begin{array}{c} \text{K}\;=\;\frac{\text{Q}}{\text{JAt}}\;=\;\frac{\text{Q}}{\text{7}.\text{5Ht}} \end{array}$$ (1)

$$\begin{array}{c} \text{H}\;=\;\text{4}\;+\;\text{h}\;+\;{\text{h}}_{\text{p}} \end{array}$$ (2)

Where: K — coefficient of permeability, cm/s

Q — volume of seepage within the test duration t, cm^3^

J — hydraulic gradient, dimensionless

A — cross-sectional area of soil sample, cm^2^; (adopted as 30 cm^2^)

t — test duration, s

H — total head, cm

h — scale reading in water tank, cm

h_p_— water column height converted from air pressure in water tank, cm

#### 2.3.2. Direct shear test.

**Fig 6:**
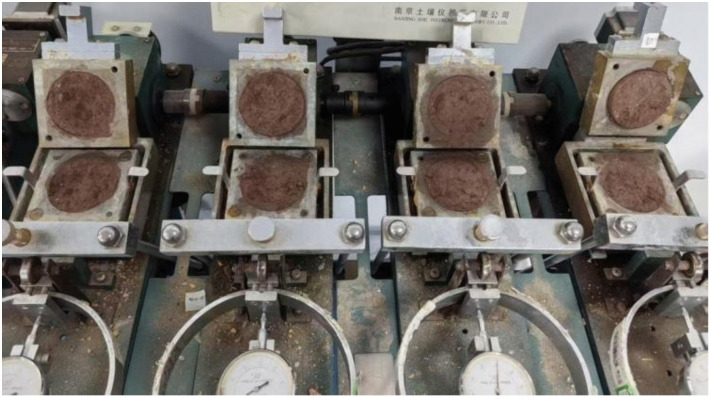
Photos of the sample direct shear test.

A ZJ strain-controlled quadruple direct shear apparatus was adopted to test shear strength indicators of six groups of specimens, as shown in Fig 6. Standard ring knife samples with height of 4 cm and cross-sectional area of 30 cm² were prepared for shear tests under normal pressure. After shear failure, the shear strength indexes of each sample were calculated and summarized.

#### 2.3.3. Micro-CT scanning test.

For the CT scanning of Sample 2#, the nanoVoxel-4200 experimental equipment for non-coal open-pit mine disaster prevention and control was adopted. This equipment is a non-destructive 3D holographic microscopic imaging device with ultra-high resolution.

The nanoVoxel-4200 employs a unique X-ray optical microscopic imaging technology. By capturing X-ray perspective images from multiple angles and combining computer-based 3D digital reconstruction, it provides high-resolution 3D images of the complex internal structure of the sample. This enables sub-micron-scale digital 3D characterization of the internal microstructure and analysis of constituent material properties.

## 3. Test results

### 3.1. Permeation test results

The permeability test results of the prepared samples are shown in Table 4.

**Table 4:** The permeability test results of the prepared samples.

Sample ID	h/cm	h_p_/cm	H/cm	t/s	Q/cm^3^	K/cm/s
1#	10	50	64	7.9	2.0	5.3e-4
2#	10	50	64	13020.8	2.0	3.2e-7
3#	10	50	64	7575.8	2.0	5.5e-7
4#	10	50	64	21929.8	2.0	1.9e-7
5#	10	50	64	29761.9	2.0	1.4e-7
6#	10	50	64	37878.8	2.0	1.1e-7

The test results indicated that the raw tailing sample has the highest permeability coefficient, which is 5.3 × 10 ⁻ ⁴ cm/s. In contrast, Samples 2# to 6# are all based on 30 kg of tailing, mixed with 3 kg of sodium silicate, 35 kg of water, and different amounts of cement and bentonite; their permeability coefficients significantly decreased to 1.1 × 10 ⁻ ⁷–5.5 × 10 ⁻ ⁷ cm/s, and the anti-seepage performance has been remarkably improved.

When the bentonite dosage was fixed at 8 kg, the hydraulic conductivity rose sequentially from 1.9 × 10 ⁻ ⁷ cm/s to 3.2 × 10 ⁻ ⁷ cm/s and finally to 5.5 × 10 ⁻ ⁷ cm/s as cement content increased from Sample 4# to Sample 2# and Sample 3#. This trend demonstrated that within the test mix proportion range, elevated cement dosage weakened the anti-seepage capacity. Excess cement hydration products generate relatively large intergranular pores and interconnected seepage pathways, counteracting the pore-filling effect of C-S-H gel.

With the cement dosage kept constant at 20 kg, increasing bentonite dosage from Sample 2# to Sample 5# and then Sample 6# led to a gradual reduction in permeability coefficient from 3.2 × 10 ⁻ ⁷ cm/s to 1.4 × 10 ⁻ ⁷ cm/s and further to 1.1 × 10 ⁻ ⁷ cm/s.The increase in bentonite dosage continuously improved the anti-seepage capacity of composite specimens.Montmorillonite in sodium bentonite absorbed water and swelled to fill micro-connected pores, effectively blocking internal seepage paths of the material.

Within the mix proportion range of this experiment, cement weakens the anti-seepage performance of the composite material, while bentonite enhances it.

### 3.2. direct shear test results

Photos of the sample direct shear test are shown in Fig 6, and the direct shear test results of the samples are presented in Table 5.

**Table 5:** The direct shear test results of the samples.

Sample ID	c/kPa	φ/°
1#	25.1	21.9
2#	152.1	35.6
3#	345.2	39.1
4#	63.2	30.2
5#	157.8	38.4
6#	182.2	38.9

The test results showed that Sample 1# (undisturbed tailing sample) has a cohesion of 25.1 kPa and an internal friction angle of 21.9°, showing relatively low shear strength; in contrast, Samples 2# to 6# (with cement and bentonite added) exhibit a significant improvement in shear strength.

Further analysis reveals that the strength of the anti-seepage material is positively correlated with both cement and bentonite content: With the bentonite dosage kept constant at 8 kg for Samples 4#, 2# and 3#, increasing cement dosage from Sample 4# to Sample 2# and further to Sample 3# led to a sequential rise in cohesion from 63.2 kPa to 152.1 kPa and further to 345.2 kPa, alongside a gradual increase in internal friction angle from 30.2° to 35.6° and further to 39.1°,which indicating that the shear strength shows a stepped increase with the increase in cement content. Increased cement generates abundant C-S-H hydration gel to cement tailing particles and fill intergranular voids, significantly improving the cohesive force and friction characteristics of the matrix.

With the cement dosage kept constant at 20 kg, increasing bentonite dosage from Sample 2# to Sample 5#and further to Sample 6# led to a sequential rise in cohesion from 152.1 kPa to 157.8 kPa and further to 182.2 kPa, accompanied by a gradual growth in internal friction angle from 35.6° to 38.4° and further to 38.9°which indicating that shear strength increases steadily with increasing bentonite content. Swollen bentonite enhances the interfacial bonding between solid particles and optimizes the internal packing structure, resulting in a slight improvement in shear strength.

Comparing the two variables, the change in cement content has a more pronounced effect on improving the shear strength of the samples.

### 3.3. Micro-CT scanning test results

The scanning parameters of this experiment are shown in Table 6.

**Table 6:** The scanning parameters of CT.

Sample ID	Scanning Voltage U(V)	Scanning Current I (mA)	Resolution(μm)）	Penetration Rate(%)	Acquisition Frame Rat(FPS)
2#	250	330	30.3	16.6	1080

**Fig 7:**
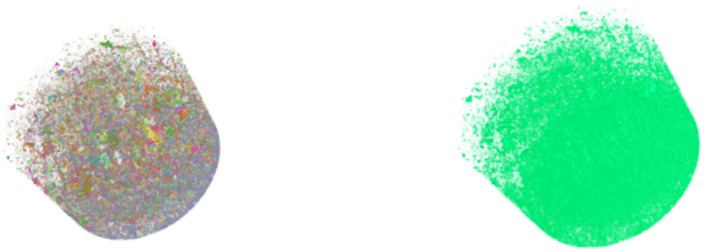
3D pore reconstruction of the specimen.

The 3D pore image of the sample is shown in Fig 7. The CT scanning results revealed that the specimen only had a porosity of 1.94%, indicating extremely low porosity, which accounts for its excellent anti-seepage performance and high strength.

## 4. Discussion

### 4.1. Mechanism

The anti-seepage performance of this composite material results from the combined effect of cementitious products generated by chemical reactions and physical pore structure densification, with various components cooperatively forming a solidified body with low permeability. The chemical reactions between the components are mainly the generation of cementitious products and ion exchange, the core of which is to form a continuous hydration product network and block seepage channels.

The core hydration reactions of Portland cement originate from four types of clinker minerals. Among them, the hydration of tricalcium silicate (C_3_S) and dicalcium silicate (C_2_S) serves as the main pathway for generating cementitious products, while the hydration of tricalcium aluminate (C_3_A) and tetracalcium aluminoferrite (C_4_AF) assists in enhancing structural compactness.


$$\begin{array}{c} \text{6CaO}\cdot \text{2}{\text{SiO}}_{\text{2}}\;+\;\text{6}{\text{H}}_{\text{2}}\text{O }\to \text{ 3CaO}\cdot \text{2}{\text{SiO}}_{\text{2}}\cdot \text{3}{\text{H}}_{\text{2}}\text{O }+\text{ 3Ca }{\left(\text{OH}\right)}_{\text{2}} \end{array}$$ (3)

$$\begin{array}{c} \text{4CaO}\cdot \text{2}{\text{SiO}}_{\text{2}}\;+\;\text{4}{\text{H}}_{\text{2}}\text{O }\to \text{ 3CaO}\cdot \text{2}{\text{SiO}}_{\text{2}}\cdot \text{3}{\text{H}}_{\text{2}}\text{O }+\text{ 1Ca }{\left(\text{OH}\right)}_{\text{2}} \end{array}$$ (4)

The main hydration reactions of Portland cement are shown in Equations 3 and 4. Among them, tricalcium silicate (C₃S) accounts for 50%−70% of cement, has a fast hydration rate, and makes a significant contribution to the early strength of cement. Dicalcium silicate (C₂S) accounts for 15%−30% of cement, undergoes slow hydration, and contributes to the late-stage strength of cement. The generated C-S-H (calcium silicate hydrate) is a cementitious material that can significantly fill pores and improve the material’s impermeability, while Ca(OH)₂ (calcium hydroxide) provides an alkaline environment for subsequent reactions.

The chemical composition of water glass is Na₂O·nSiO₂ (where n is the modulus, usually ranging from 2.8 to 3.2). It reacts with Ca(OH)₂ in an alkaline environment to generate additional C-S-H gel and sodium hydroxide (NaOH), which significantly accelerates the cementation process and densifies the structure.


$$\begin{array}{c} {\text{Na}}_{\text{2}}\text{O}\cdot \text{n}{\text{SiO}}_{\text{2}}\;+\text{ Ca }{\left(\text{OH}\right)}_{\text{2}}\;+\;(\text{2n}-\text{1}){\text{H}}_{\text{2}}\text{O }\to \text{ CaO}\cdot \text{n}{\text{SiO}}_{\text{2}}\cdot \text{2n}{\text{H}}_{\text{2}}\text{O }+\text{ 2Na }(\text{OH}) \end{array}$$ (5)

The main mineral of sodium bentonite is montmorillonite, which has a layered crystal structure with exchangeable cations (mainly Na^+^) between the layers. In aqueous solution, the Na+ between the montmorillonite layers will undergo ion exchange with the Ca^2+^ generated by cement hydration, improving the hydration and swelling capacity of bentonite. After ion exchange, the hydrophilicity of bentonite is enhanced. When it absorbs water, its volume expands, which can block tiny pores and strengthen the anti-seepage effect.

**Fig 8:**
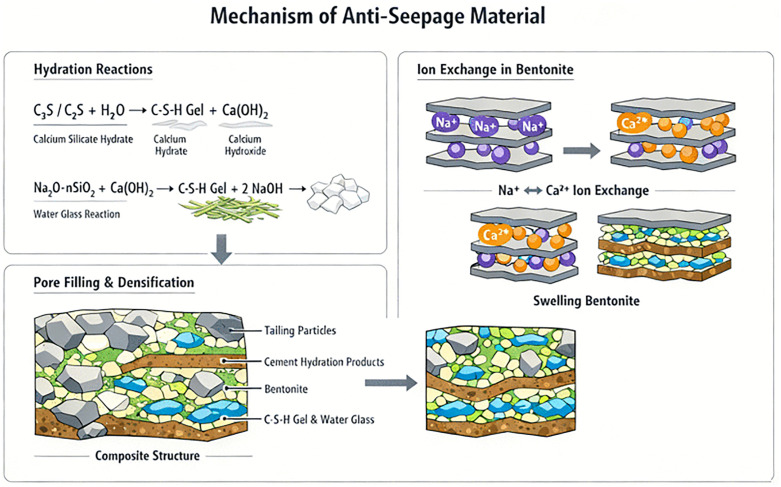
The reaction results of the composite material.

Tailing, used as aggregates, have a particle size distribution that can form a dense packing with cement and bentonite particles: large tailing particles form a skeleton, cement and bentonite particles fill the gaps between tailing, and water glass and cement hydration products fill the tiny pores between particles, achieving multi-level pore filling and significantly reducing the connected porosity of the material.The reaction results of the composite material are shown in Fig 8.

Both the strength and permeability coefficient of the composite materials depended on the regulation of structural compactness The core influencing components include calcium silicate hydrate (C-S-H) gel, expanded montmorillonite, and ettringite/calcium aluminate hydrate.

Among them, C-S-H gel is the core cementitious material. It can not only directly improve the cohesion and internal friction angle of the material by cementing particles and filling pores — for example, when the cement dosage increases, the yield of C-S-H gel increases, and the cohesion rises from 63.2 kPa to 345.2 kPa in the direct shear test — but also fill the connected pores between particles to block seepage channels. Although the increase in cement dosage weakens the anti-seepage performance in Samples 4#-2#-3# C-S-H gel remains the core factor for anti-seepage.

After ion exchange, expanded montmorillonite absorbs water and swells. On one hand, it can fill micro-pores and reduce loose areas inside the structure to improve integrity, thereby indirectly enhancing shear strength. For instance, in Samples 2#-5#-6#, the increase in bentonite dosage leads to a rise in cohesion from 152.1 kPa to 182.2 kPa. On the other hand, it can block micro-seepage channels not covered by cementitious products and reduce the connected porosity of the material; specifically, the increase in bentonite dosage in Samples 2#-5#-6# causes the permeability coefficient K to decrease from 3.2 × 10 ⁻ ⁷ to 1.1 × 10 ⁻ ⁷ cm/s.

Ettringite calcium aluminate hydrate can improve structural compactness by filling micro-pores, thereby assisting in enhancing strength. Especially when the cement dosage is relatively high, more ettringite is generated from C₃A, resulting in a more significant improvement in compactness — for example, the internal friction angle (φ) of Sample 3# reaches 39.1°.

From a theoretical perspective, this study delivers two core theoretical contributions to the research system of tailings-based composite anti-seepage materials.

First, a multi-stage synergistic pore densification theoretical framework is proposed for high-content fine tailings composite barriers. The framework quantitatively clarifies the hierarchical filling logic: tailings particles form the primary skeleton, cement hydration C-S-H gel fills inter-particle voids, sodium bentonite montmorillonite blocks micro seepage channels after ion exchange, and sodium silicate alkali-activated products fill residual microcracks. It systematically reveals the competitive regulation law of cement and bentonite on hydraulic conductivity and shear strength, which compensates for the lack of quantitative theoretical analysis of dual-factor synergistic mechanisms in existing tailings barrier material studies.

Second, this paper improves the theoretical understanding of the coupling relationship between ion exchange and seepage resistance of sodium bentonite under high alkaline tailings-cement environments. Previous studies only separately discussed bentonite swelling or cement hydration, while this work reveals that Ca^2+^ released by cement hydration triggers ion exchange between bentonite interlayer cations, further amplifying montmorillonite expansion capacity. The established correlation between cation exchange degree, pore connectivity and long-term anti-seepage performance supplements the theoretical basis for the durability evaluation of solid waste composite cutoff walls.

In engineering theory, the established quantitative correlation curve between component dosage, pore structure index and macro hydraulic-mechanical indicators can provide a theoretical calculation reference for the mix proportion design of tailings vertical anti-seepage walls, breaking the over-reliance on empirical mixing ratios in traditional cement-bentonite wall design.

## 5. Conclusions

This study developed a fine-grained tailing composite anti-seepage material using tailing sand as the main aggregate, optimized its mix proportion via single-factor controlled variable tests, and investigated its performance through permeability/shear strength tests and CT microscopic analysis.

This study developed a composite anti-seepage barrier material dominated by fine-grained tailings, and systematically clarified the independent and coupled effects of cement and sodium bentonite on hydraulic conductivity and shear strength through permeability tests, direct shear tests and Micro-CT microscopic characterization. Pure tailings exhibited extremely high permeability of 5 × 10^-4 cm^/s, while multi-component compound modification reduced the hydraulic conductivity to 1 × 10^-7 cm^/s ~ 5.5 × 10^-7 cm^/s. Cement significantly improved shear strength at the cost of slightly increasing permeability, whereas sodium bentonite continuously optimized anti-seepage performance with mild strength promotion. Micro-CT results confirmed the optimal specimen only had a porosity of 1.94%, which originated from multi-stage synergistic densification including tailings skeleton support, C-S-H gel filling and bentonite swelling pore-blocking.

The present work delivers clear theoretical and practical implications. The proposed multi-stage pore densification framework supplements quantitative theoretical analysis for tailings-based composite barriers and enriches the coupling theory of bentonite ion exchange and seepage resistance under alkaline cement-tailings environments. Practically, this material realizes large-volume on-site tailings recycling, balances low permeability and reliable mechanical performance, and can provide direct mix proportion guidance for tailings pond vertical cutoff walls to reduce engineering raw material costs and solid waste stockpiling pressure.

Several limitations remain for further exploration. Only short-term static hydraulic and mechanical tests under normal temperature were conducted, lacking long-term durability experiments such as dry-wet cycles, freeze-thaw cycles and chemical erosion. Single-factor tests were adopted without multi-factor interaction optimization, and microscopic characterization only relied on Micro-CT without supplementary SEM analysis. All tests were completed in laboratory scale, and field construction pilot verification is required to evaluate practical workability and long-term service stability.

## Supporting information

S1 File Include minimal experimental data set for the study on fine-grained tailings composite anti-seepage material, including raw material physical test data, specimen mix proportions, permeability test results, direct shear strength data and Micro-CT scanning parameters. All laboratory tests and measurement data were supplied by the State Key Laboratory of Metal Mine Mining Safety and Disaster Prevention and Control.
(DOCX)
