# Supplementary material for: Study on mix proportion and mechanism of fine-grained tailing composite anti-seepage material
Source: PLoS One. 2026 Aug 11;21(8):e0355719. doi: 10.1371/journal.pone.0355719 (PMC13460562; doi:10.1371/journal.pone.0355719)
Supplement: Supplementary file 1 [file pone.0355719.s001.docx]

Minimal Data

This minimal data set corresponds to the paper 'Study on Mix Proportion and Mechanism of Fine-grained Tailing Composite Anti-seepage Material' and contains all core quantitative experimental data required to support the paper’s material performance analysis, chart plotting and microscopic mechanism interpretation. The data set is divided into four interrelated subsets derived from systematic laboratory tests:

1. Raw material basic characteristic data set. It covers fundamental physical indicators and particle size distribution of full fine tailings, mineral and chemical composition parameters of 42.5R ordinary Portland cement, as well as XRD mineral phase and montmorillonite content test data of sodium bentonite, which lay a basic parameter foundation for mix proportion design.

2. Specimen mix proportion data set. Six groups of specimens are designed with raw tailings as the control group. The fixed mass of full tailings, sodium silicate and water is adopted, while cement and sodium bentonite dosages are set as single variable factors, recording the absolute mass and mass percentage of each raw material for all specimens.

3. Macro hydraulic-mechanical performance test data set. It includes complete raw measurement data of low-permeability permeability tests, such as water tank scale reading, converted air pressure water column height, total water head, test duration, seepage volume and final permeability coefficient of each sample. Meanwhile, the data set collects shear strength indicators (cohesion and internal friction angle) obtained from quadruple strain-controlled direct shear tests, which quantitatively reflect the dual influence of cement and bentonite on impermeability and mechanical stability.

4. Microscopic Micro-CT scanning data set. It records complete scanning equipment parameters of the optimal specimen, including voltage, current, spatial resolution, penetration rate and frame rate, as well as the integral porosity result of the 3D reconstructed pore structure. Supplementary chemical reaction formulas of cement hydration, sodium silicate alkali activation and bentonite cation exchange are also provided to support the multi-component synergistic densification mechanism analysis.

All redundant repeated measurement data are eliminated, and only the minimal valid data closely matched with the paper’s research conclusions are retained. This data set can be reused for the mix proportion optimization of tailing solid waste-based anti-seepage materials, quantitative correlation analysis between raw material dosage, pore microstructure and macro engineering performance, and comparative experimental research on mine tailings pond vertical cutoff wall materials.

**Table 1. Physical Properties of tailing Specimen**

| w(%) | ρ(g/cm^3^) | ρ_d(_g/cm^3^_)_ | Gs | e | I_p_（%） |
| --- | --- | --- | --- | --- | --- |
| 12.6 | 1.03 | 0.91 | 3.02 | 2.301 | 8.6 |

**Table 2. Particle Size Range**

| Particle size Range（mm） | 2~0.5 | 0.5~0.25 | 0.25~0.075 | <0.075 |
| --- | --- | --- | --- | --- |
| Percentage（%） | 0.9 | 4.9 | 21.0 | 73.2 |

**Table 3. The permeability test results of the prepared samples**

| Sample ID | h/cm | h_p_/cm | H/cm | t/s |
| --- | --- | --- | --- | --- |
| 1# | 10 | 50 | 64 | 7.9 |
| 2# | 10 | 50 | 64 | 13020.8 |
| 3# | 10 | 50 | 64 | 7575.8 |
| 4# | 10 | 50 | 64 | 21929.8 |
| 5# | 10 | 50 | 64 | 29761.9 |
| 6# | 10 | 50 | 64 | 37878.8 |

**Table 4. The direct shear test results of the samples**

| Sample ID | c/kPa | φ/° |
| --- | --- | --- |
| 1# | 25.1 | 21.9 |
| 2# | 152.1 | 35.6 |
| 3# | 345.2 | 39.1 |
| 4# | 63.2 | 30.2 |
| 5# | 157.8 | 38.4 |
| 6# | 182.2 | 38.9 |

**Table 5. The scanning parameters of CT**

| Sample ID | Scanning Voltage U（V） | Scanning Current I  （mA) | Resolution（μm） | Penetration Rate(%） | Acquisition Frame Rat（FPS） |
| --- | --- | --- | --- | --- | --- |
| 2# | 250 | 330 | 30.3 | 16.6 | 1080 |

All laboratory tests were completed and all experimental data were provided by the State Key Laboratory of Metal Mine Mining Safety and Disaster Prevention and Control. Please contact us if you have any questions regarding the data set or require further supplementary test information.
